# Development and Evaluation of MR-Based Radiogenomic Models to Differentiate Atypical Lipomatous Tumors from Lipomas

**DOI:** 10.3390/cancers15072150

**Published:** 2023-04-05

**Authors:** Sarah C. Foreman, Oscar Llorián-Salvador, Diana E. David, Verena K. N. Rösner, Jon F. Rischewski, Georg C. Feuerriegel, Daniel W. Kramp, Ina Luiken, Ann-Kathrin Lohse, Jurij Kiefer, Carolin Mogler, Carolin Knebel, Matthias Jung, Miguel A. Andrade-Navarro, Burkhard Rost, Stephanie E. Combs, Marcus R. Makowski, Klaus Woertler, Jan C. Peeken, Alexandra S. Gersing

**Affiliations:** 1 Department of Radiology, Klinikum Rechts der Isar, Technische Universität München, Ismaninger Straße 22, 81675 Munich, Germany; 2 Department of Radiation Oncology, Klinikum Rechts der Isar, Technische Universität München, Ismaninger Straße 22, 81675 Munich, Germany; 3 Department of Informatics, Bioinformatics and Computational Biology—i12, Technische Universität München, Boltzmannstr. 3, 85748 Munich, Germany; 4 Institute of Organismic and Molecular Evolution, Johannes Gutenberg University Mainz, Hanns-Dieter-Hüsch-Weg 15, 55128 Mainz, Germany; 5 Department of Diagnostic and Interventional Neuroradiology, University Hospital Munich (LMU), Marchioninistrasse 15, 81377 Munich, Germany; 6 Department of Radiology, University Hospital Munich (LMU), Marchioninistrasse 15, 81377 Munich, Germany; 7 Department of Plastic Surgery, University Hospital Freiburg, University of Freiburg, Hugstetterstraße 55, 79106 Freiburg im Breisgau, Germany; 8 Institute of Pathology, Klinikum Rechts der Isar, Technische Universität München, Ismaninger Straße 22, 81675 Munich, Germany; 9 Department of Orthopedics and Sport Orthopedics, Klinikum Rechts der Isar, Technische Universität München, Ismaninger Straße 22, 81675 Munich, Germany; 10 Department of Radiology, University Hospital Freiburg, University of Freiburg, Hugstetterstraße 55, 79106 Freiburg im Breisgau, Germany; 11 Helmholtz Zentrum München, Deutsches Forschungszentrum für Umwelt und Gesundheit, Institute of Radiation Medicine Neuherberg, 85764 Munich, Germany; 12 Deutsches Konsortium für Translationale Krebsforschung (DKTK), Partner Site Munich, 69120 Heidelberg, Germany

**Keywords:** radiomics, machine learning, soft-tissue sarcomas, radiology, MRI

## Abstract

**Simple Summary:**

Differentiating atypical lipomatous tumors from lipomas on MR images is a challenging task due to similar imaging characteristics. Given these challenges, it would be highly beneficial to develop a reliable diagnostic tool, thereby minimizing the need for invasive diagnostic procedures. Therefore, the aim of this study was to develop and validate radiogenomic machine-learning models to predict the MDM2 gene amplification status in order to differentiate between ALTs and lipomas on preoperative MR images. The best machine-learning model was based on radiomic features from multiple MR sequences using a LASSO algorithm and showed a high discriminatory power to predict the MDM2 gene amplification. Due to the varying settings in which patients with lipomatous tumors present, this model may enhance the clinical diagnostic workup.

**Abstract:**

Background: The aim of this study was to develop and validate radiogenomic models to predict the MDM2 gene amplification status and differentiate between ALTs and lipomas on preoperative MR images. Methods: MR images were obtained in 257 patients diagnosed with ALTs (*n* = 65) or lipomas (*n* = 192) using histology and the MDM2 gene analysis as a reference standard. The protocols included T2-, T1-, and fat-suppressed contrast-enhanced T1-weighted sequences. Additionally, 50 patients were obtained from a different hospital for external testing. Radiomic features were selected using mRMR. Using repeated nested cross-validation, the machine-learning models were trained on radiomic features and demographic information. For comparison, the external test set was evaluated by three radiology residents and one attending radiologist. Results: A LASSO classifier trained on radiomic features from all sequences performed best, with an AUC of 0.88, 70% sensitivity, 81% specificity, and 76% accuracy. In comparison, the radiology residents achieved 60–70% accuracy, 55–80% sensitivity, and 63–77% specificity, while the attending radiologist achieved 90% accuracy, 96% sensitivity, and 87% specificity. Conclusion: A radiogenomic model combining features from multiple MR sequences showed the best performance in predicting the MDM2 gene amplification status. The model showed a higher accuracy compared to the radiology residents, though lower compared to the attending radiologist.

## 1. Introduction

Lipomatous tumors are the most common neoplasms encountered by physicians and the most frequent soft-tissue tumors of the extremities [1]. Of these, 40 to 45% are benign adipocytic tumors (lipomas) or atypical lipomatous tumors (ALTs) [2,3,4,5]. Lipomas only require treatment if the mass effect causes symptoms such as pain or functional disorders [6]. ALTs may show locally aggressive growth and may dedifferentiate into high-grade sarcomas [7,8,9,10]. Therefore, ALTs are typically resected [11]. Histopathological differentiation relies on the detection of atypical hyperchromatic nuclei and the immunohistochemical evaluation of the molecular analysis of the mouse double minute 2 (MDM2) gene [12]. However, the detection of these atypical hyperchromatic cells can be challenging since they are frequently scattered throughout the lesion, and detection is often complicated by fibrous septa, subsequently requiring a careful analysis of the entire tumor [12,13,14]. Previous studies have shown that the MDM2 amplification status is the most accurate marker to differentiate ALTs and lipomas, and there is a tendency towards sampling errors if the MDM2 status is not determined [12,15,16,17]. Unfortunately, the majority of MR imaging studies differentiating ALTs from lipomas did not include a molecular analysis, or only performed a molecular analysis in a subset of patients [6,14,18,19].

MR imaging is the standard imaging modality for the assessment of soft-tissue tumors due to its excellent soft-tissue contrast [20,21,22]. Specific imaging features such as the tumor size, tumor location, presence of thick septa, and amount of contrast uptake can be used to differentiate ALTs from lipomas [6,13,18,19,23]. However, since there is a substantial overlap between these imaging features in both tumor types, differentiating ALTs from lipomas is a challenging task. Moreover, previous studies of systematic radiologic readings have reported relatively low inter-observer reproducibility, with a kappa agreement ranging from 0.17 to 0.42 [13,19,24]. Given these challenges, it would be highly beneficial to develop a reliable diagnostic tool to differentiate ALTs from lipomas on preoperative MR images, thereby minimizing the need for invasive diagnostic procedures.

Machine-learning techniques, including imaging-based radiomics, permit a non-invasive detailed analysis of a tumor phenotype by using a quantitative imaging feature analysis [25,26]. However, one of the main challenges of radiomic models includes reproducibility in different datasets [27,28]. Therefore, the aim of this study was to develop and validate radiogenomic machine-learning models based on multiparametric MR examinations to predict the MDM2 gene amplification status in order to differentiate between ALTs and lipomas on preoperative MR images. The models were evaluated using an independent external cohort for testing and were compared to the performance of radiologists.

## 2. Materials and Methods

The local institutional review boards approved this retrospective multi-center study (ethics committee 666/21 S) The study was performed in accordance with our institutional ethic guidelines and the 1964 Declaration of Helsinki and its later amendments. Written and informed consent was waived for this retrospective anonymized analysis.

### 2.1. Datasets

**Figure 1:**
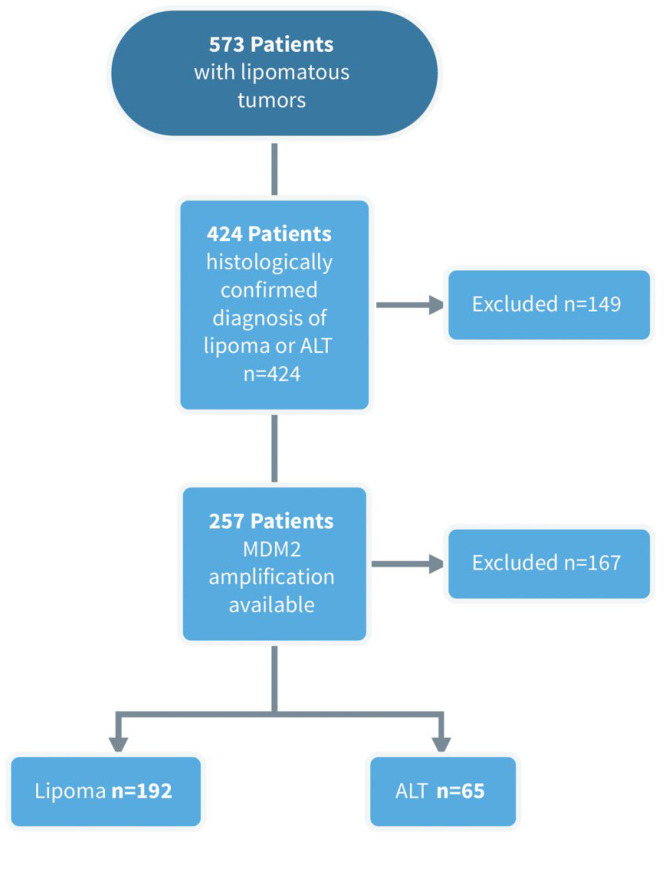
Subject selection flowchart. ALT = atypical lipomatous tumor; MDM2 = murine double minute.

We retrospectively reviewed the records of all patients with lipomatous tumors in the upper or lower extremities or trunk that had surgery performed at our sarcoma referral center between 2010 and 2021 (*n* = 573). Of these, 424 patients had a histologically confirmed diagnosis of a lipoma or an ALT. The MDM2 amplification status, determined by fluorescence in situ hybridization (FISH) of the MDM2 gene locus, was available for *n* = 257 patients. Patients without an MDM2 amplification status were excluded. Therefore, in the final dataset, both the histology and the MDM2 gene amplification status were available for all patients. Two senior pathologists specializing in the analysis of soft-tissue tumors provided a final consensus diagnosis based on the MDM2 gene amplification status and histology according to the World Health Organization criteria. The patient selection process is shown in Figure 1.

In addition, an external test set was obtained from a further sarcoma referral center, the University Hospital of Freiburg (M1), for final independent testing and geographical validation. The external test set included patients with a diagnosis of a lipoma or an ALT confirmed by their histology and MDM2 amplification status.

### 2.2. MR Imaging Protocol and Image Segmentation

Pre-operative MR images were acquired using 3 or 1.5 Tesla scanners. Sequences were acquired in at least two planes that were oriented along the short and longitudinal axes of the long articulating bone(s). The protocols included a T2-w turbo spin echo (TSE) sequence (T2w), a T1-w TSE sequence (T1w), and a fat-saturated T1-w TSE sequence after the administration of a contrast agent (T1fsgd). Detailed information on the acquisition parameters is provided in Supplementary Material Table S1.

To define the volumes of interest (VOIs), tumor segmentations were performed manually by two radiology residents (S.C.F. and G.C.F.) using the open-source software 3D Slicer (3D Slicer, Version 4.8, stable release) and extracted as Neuroimaging Informatics Technology Initiative (NIfTI) label maps for further analysis. Multiple delineations were performed by S.C.F. and G.C.F. in 20 randomly selected patients to account for inter-reader variability.

### 2.3. Radiomic Feature Extraction and Machine-Learning Model Development

All preprocessing steps and radiomic feature extractions were conducted in accordance with the Imaging Biomarker Standardization Initiative guidelines [29] using the Python package PyRadiomics (version 2.2) implemented in Python (3.7), as previously described [30]. Image discretization was conducted using a bin width of 10 to achieve a bin count between 16 and 128, as recommended by the pyradiomics documentation [31]. Image intensity normalization was achieved via redistributing the image at the mean with a standard deviation and a scale of 100. Bspline interpolation was used to perform isotropic resampling to a voxel size of 1 × 1 × 1 mm of the image and VOI mask. A total of 104 features were extracted from the original image of each sequence within the segmented label map (resulting in a total of 312 radiomic features), including first-order features, shape features, and texture features. The latter comprised “gray-level co-occurrence matrix” features, “gray-level size-zone matrix” features, “gray-level run-length matrix” features, “neighboring gray-tone difference matrix” features, and “gray-level dependence matrix” features. No features were extracted from filtered versions of the image due to a missing IBSI consensus. A detailed list of all extracted features is provided in Supplementary Material Table S2. Feature values were transformed to a common scale using min–max normalization in order to conserve their original distribution in the [0,1] range. Data normalization was performed prior to splitting the data into training and testing groups due to the batch harmonization step requirements. Nonparametric ComBatBatch harmonization was applied to account for the variability introduced by different MR scanners, as described previously [30]. Clinical features such as age, sex, and body region of the tumor (torso/head, upper extremity, or lower extremity) were also included. Categorical features were encoded into dummy numeric arrays using one hot encoder. All radiomic features susceptible to segmentation variations were excluded using a threshold intraclass correlation coefficient (ICC 3,1) of 0.8. This statistic resulted in 5, 15, and 4 radiomic features that were excluded from the T1w, T2w, and T1fsgd sequences, respectively. ICC 3,1 was chosen, as the raters were not rated as representative of a defined rater group due to their differing extents of training.

**Figure 2:**
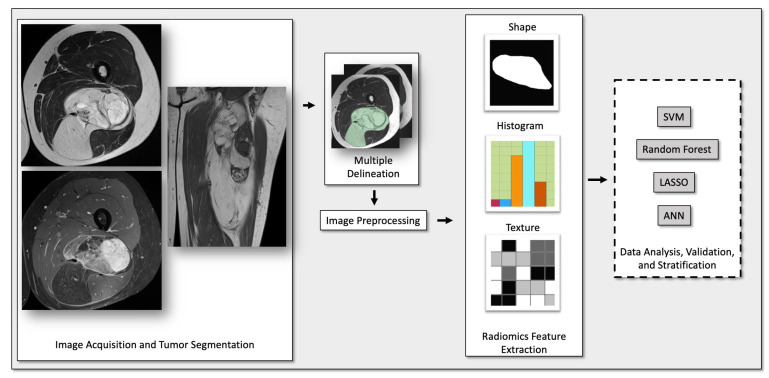
Radiomic workflow. Abbreviations: SVM, support vector machine; LASSO, least absolute shrinkage and selection operator; ANN, artificial neural network.

An estimate of the number of reduced features to use was calculated using a principal component analysis (PCA) with 95% of data variance: 11 to 13 features for the individual sequences (T1w, T2w, and T1fsgd) and 19 to 21 features for the combined features of all sequences. Each respective number of features was selected using minimum redundancy–maximum relevance (MRMR). Synthetic minority over-sampling and random under-sampling of the majority class were used to counteract the class imbalance. The ratios were tuned to find an optimal balance between data augmentation and data discard, with ratios of 0.5–0.6:1 after SMOTE and 0.6–0.8:1 after the random under-sampling of the majority class. The remaining class imbalance was handled by using balanced accuracy as the optimization criteria during hyperparameter optimization. Four machine-learning algorithms were implemented and compared in their performance: the support vector machine (SVM), the random forest classifier (RFC), the least absolute shrinkage and selection operator (LASSO; built from a stochastic gradient descent classifier), and a fully connected, feedforward artificial neural network (ANN; multilayer perceptron classifier). A flow chart of the data processing and analysis of the radiomic features can be found in Supplementary Material Figure S1. For each algorithm, models were developed by (i) using demographic information only, (ii) using radiomic features for each individual sequence (T1w, T2w, or T1fsgd), (iii) using the radiomic features of all sequences, and (iv) using a combination of both the radiomic features of all sequences and demographic information. An overview of the radiomic workflow is shown in Figure 2.

### 2.4. Model Optimization, Evaluation, and Statistical Analysis

Training and validation were performed using 3-fold nested cross-validation with 50 repetitions for statistical robustness, for a total of 150 averaged iterations per modeling algorithm and dataset. Hyperparameter optimization was conducted using an exhaustive grid search. This step was performed in the inner fold, after the feature selection step via MRMR, to prevent data leakage. Balanced accuracy was used as the optimization criterion to determine the best set of hyperparameters.

The performance of the models was evaluated with the area under the curve (AUC) obtained from the receiver–operator curve (ROC), plotted after averaging the yielded values. We also included the accuracy, sensitivity, and specificity as the output measures. For an unbiased evaluation, a final cross-validation step was implemented by selecting the best values obtained from the internal dataset before evaluating the performance on the external dataset. Stochastic gradient descent was used to calculate the probability of each class prediction. Calculations of model metrics were performed using scikit-learn (version 1.0.2). 

For comparison, MR images of the external test set were rated independently by three radiology residents (I.L., S.C.F., and G.C.F., with 2, 3, and 5 years of experience, respectively) and one musculoskeletal imaging fellowship-trained radiologist (A.S.G., with 10 years of experience) experienced in musculoskeletal tumor imaging. All readers were blinded to all clinical and histopathological findings.

## 3. Results

### 3.1. Study Subjects

A total of 257 patients were included in the internal dataset (192 lipomas, 65 ALTs; age, 62.4 ± 14.5 years; 125 (48.6%) women). Fifty patients were included in the external dataset (30 lipomas, 20 ALTs; age, 60.6 ± 12.5 years; 22 (44%) women). All patients had a lipomatous tumor in one of the following six regions: chest, back, neck, leg, arm, hand, or foot. In both datasets, the highest number of patients had a tumor located in the leg (143/257 in the internal dataset and 27/50 in the external dataset), while the fewest number of patients had a tumor located in the foot (two in the internal dataset and none in the external dataset). Table 1 provides an overview of the subject characteristics.

### 3.2. Evaluation of the Developed Machine-Learning Models

Table 2 shows the final performance of the developed models on the external test set using demographic information only, radiomic features only (of all sequences combined), and a combination of demographic and radiomic features. The best-performing machine-learning model was based on a LASSO algorithm using a combination of all sequences, achieving an AUC of 0.88 at 70% sensitivity and 81% specificity with an accuracy of 76% on the external test set. The feature importance table, a confusion matrix, and a boxplot of the prediction probabilities from this model can be found in Supplementary Material Table S5, Supplementary Material Figure S2, and Supplementary Material Figure S3, respectively.

The AUC and accuracy for the individual sequences were lower for most models compared to models based on the radiomic parameters from all sequences combined, with a more imbalanced sensitivity/specificity. For T1w, the LASSO algorithm yielded an AUC of 0.83 at 80% sensitivity and 43% specificity with an accuracy of 58%. For T2w, the AUC was 0.82 at 42% sensitivity and 83% specificity with an accuracy of 69%. The highest AUC (0.84) was yielded for the T1fsgd sequences, though the sensitivity and specificity were highly imbalanced at 6% and 100%, respectively, with an accuracy of 60%. The performance of the developed models for the individual sequences on the external test set is shown in Supplementary Material Table S3.

**Figure 3:**
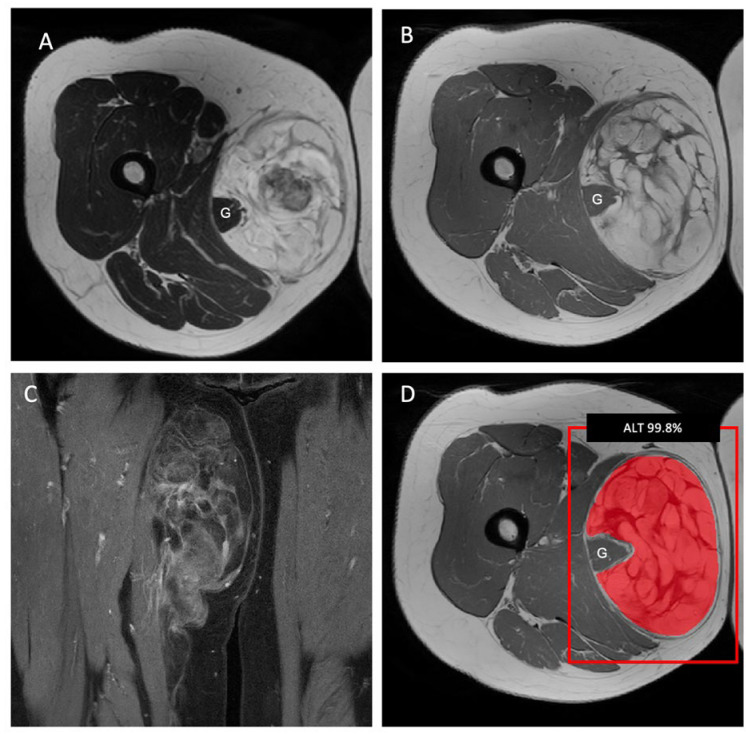
Lipomatous tumor in the medial right thigh, encasing the gracilis muscle (G). (**A**) The axial T2-weighted and (**B**) axial T1-weighted MR images show a large heterogeneous tumor with thick septa. (**C**) Septal contrast enhancement on the coronal T1-weighted images with fat saturation. (**D**) The machine-learning algorithm classified the tumor as an ALT with a probability of 99.8%. This diagnosis was confirmed by pathology and immunohistochemistry after surgical resection.

**Figure 4:**
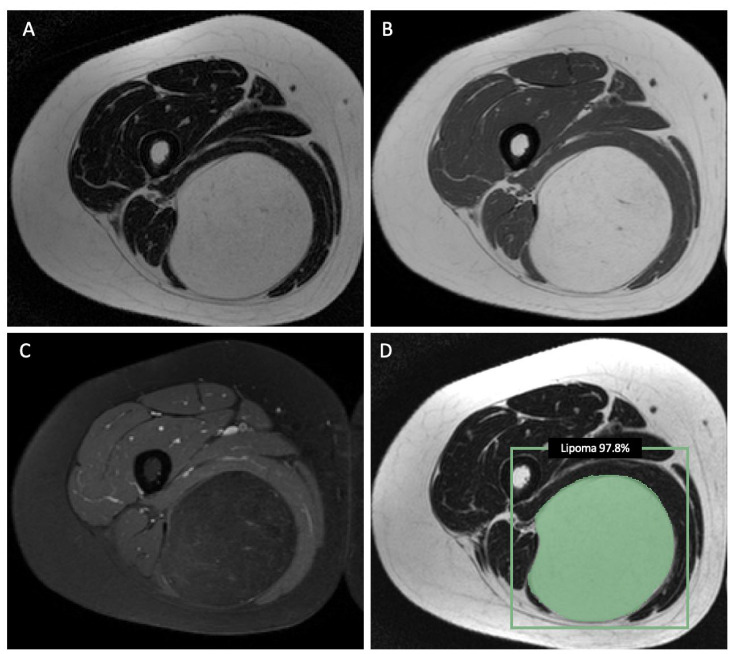
Axial T2-weighted (**A**) and T1-weighted (**B**) MR images showing a well-defined intramuscular lipomatous tumor (lipoma) in the right posterior thigh without significant contrast enhancement on the axial T1-weighted image with fat saturation (**C**). (**D**) The machine-learning model classified this tumor as a lipoma (probability of 97.8%). This was in accordance with the diagnosis made by the radiology residents and the attending radiologist.

Interestingly, combining radiomic features and demographic information as the input for the machine-learning models did not improve the performance of the LASSO algorithm to differentiate ALTs from lipomas and resulted in a decrease in the sensitivity from 70% to 40%, though the specificity increased to 100%. The averaged nested cross-validation results of the internal dataset are shown in Supplementary Material Table S4. The training parameters and source code can be found online (https://github.com/deedeedav/alt-lipoma-radiomics (accessed on 9 March 2023)). Figure 3 shows an example of an ALT with typical imaging findings encasing the right gracilis muscle, while Figure 4 shows a typical example of a well-defined intramuscular lipoma in the right posterior thigh. Both cases were identified correctly by the machine-learning model. 

### 3.3. Comparison with Radiologists

**Figure 5:**
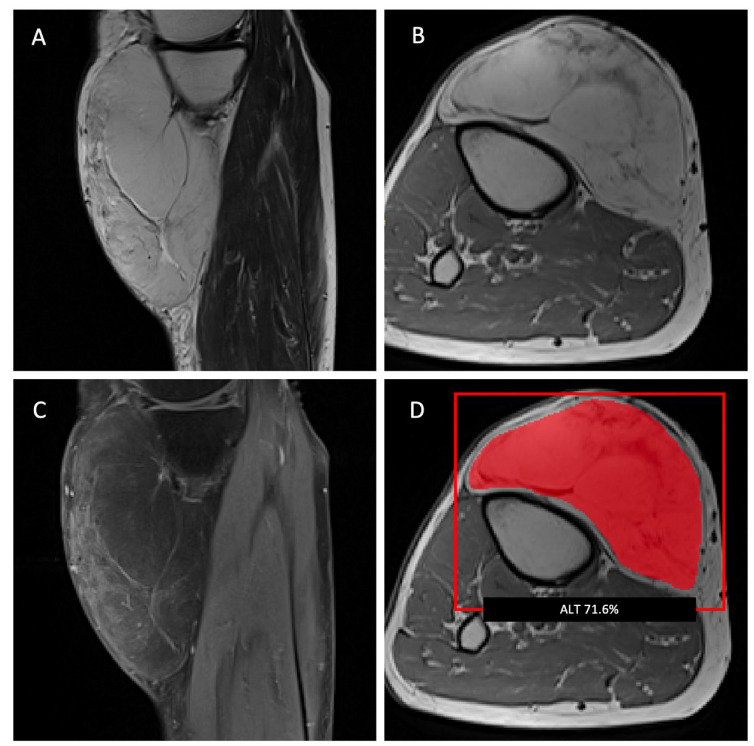
Sagittal T2-weighted (**A**) and axial T1-weighted (**B**) MR images of a lipomatous tumor located subcutaneously, anteromedial to the right proximal tibia. (**C**) A sagittal T1-weighted image with fat saturation shows a moderate septal contrast enhancement. All radiology residents classified this tumor as a lipoma, while the attending radiologist classified this tumor as an ALT. (**D**) The machine-learning algorithm also classified this tumor as an ALT with a probability of 71.6%. The diagnosis of an ALT was confirmed by pathology after surgical resection.

The results of the independent radiological readings of the external test are shown in Table 3. The radiology resident with 2 years of experience achieved an accuracy of 60%, a sensitivity of 55%, and a specificity of 63%; the resident with 3 years of experience achieved an accuracy of 70%, a sensitivity of 60%, and a specificity of 77%; and the radiology resident with 5 years of experience achieved an accuracy of 70%, a sensitivity of 80%, and a specificity of 63%. In comparison, the attending radiologist that was experienced in musculoskeletal tumor imaging achieved an accuracy of 90%, a sensitivity of 96%, and a specificity of 87%. Compared to the radiology residents, the model showed a higher accuracy and higher specificity, while the sensitivity was lower compared to the resident with 5 years of experience, but higher compared to the residents with 2 or 3 years of experience. The attending radiologist had a higher accuracy, sensitivity, and specificity. Figure 5 shows an ALT with atypical imaging findings located subcutaneously. The machine-learning model and the attending radiologist classified this tumor as an ALT, while all residents classified this tumor as a lipoma.

## 4. Discussion

In this study, machine-learning models were developed and validated to predict the amplification status of the MDM2 gene, to differentiate between atypical lipomatous tumors and lipomas on preoperative MR images, and to compare the results to the performance of radiologists using an external test set. The best-performing model was based on the combination of all MR sequences and achieved an AUC of 0.88 at 70% sensitivity and 81% specificity with an accuracy of 76%. In comparison, the accuracy of the readings by all radiology residents was lower, while the accuracy of the fellowship-trained radiologist was higher. Notably, the performance of the LASSO algorithm for each individual sequence was lower compared to the model that included all sequences (T2w, T1w, and T1fsgd), suggesting that all sequences are required for optimal discrimination.

Radiomic models for differentiating lipomas from ALTs have previously been developed in smaller patient cohorts. Leporq et al. evaluated 2D radiomic models of 40 lipomas and 41 ALTs, including one MR image slice per patient [32]. Their best-performing model achieved an accuracy of 95% at 100% sensitivity and 90% specificity using the histology as the reference standard, though no specific information regarding the MDM2 gene amplification status was included, which may have led to a false classification of ALTs as lipomas [32]. Cay et al. evaluated 45 lipomas and 20 ALTs using histology and MDM2 amplification as the gold standards [33]. They achieved an AUC of 0.987 at 96.8% sensitivity and 93.72% specificity using 1000-fold bootstrapping [33]. However, since there was no separate test set, the algorithm was likely optimized on data used for validation in another bootstrapping iteration; therefore, these results may be inaccurately high [33]. A study by Vos et al. included 116 patients (58 lipomas and 58 ALTs) and used MDM2 amplification as the reference standard [34]. Their model performance was lower compared to our study, yielding an AUC of 0.81 at 66% sensitivity and 84% specificity with an accuracy of 75%. An important limitation of these aforementioned studies is that no external validation on an independent dataset was included. Also notably, the model performance was comparatively high in studies based on smaller patient cohorts (*n* < 90). A possible explanation may be a lack of variation in smaller datasets, which could affect the reproducibility in different datasets. However, this is not clear, since no external testing was included. 

Interestingly, combining imaging parameters and clinical data did not improve the performance of most models for differentiating ALTs from lipomas, or only improved the performance marginally. While some demographic differences have been described between patients with ALTs and lipomas [23], it is likely that radiomic MR features are considerably more relevant for differentiating between these tumor types, and including parameters with less predictive power could hinder the capability of the models to identify relevant patterns. It should be noted that only a limited number of clinical features were included (age, sex, and tumor body region). Including additional clinical features may improve the predictive value of the radiomic models. Future studies could also include clinical outcome parameters to detect image-defined high-risk patients, thereby individualizing tumor treatment. 

Some limitations are pertinent to this study. Since the cohort included only patients with histopathologically confirmed tumors, this potentially introduced a selection bias. Moreover, our specialized sarcoma center typically only receives larger or atypical lipomas on referral, subsequently increasing the amount of particularly challenging lipoma cases in the dataset. We also used manual segmentations as input for the models, and developing a pipeline that includes automated segmentations would be highly beneficial. In addition, more advanced sequences such as diffusion-weighted imaging or pharmacokinetic dynamic contrast-enhanced imaging were not included in the protocol. Including these sequences could potentially improve the differentiation between ALTs and lipomas. Finally, the developed models only differentiated between ALTs and lipomas, and while this is the most challenging and clinically relevant task, further studies are warranted on the ability to distinguish among all benign and malignant lipomatous tumors. 

The advantages of the current study include its multicenter design, which allowed the evaluation of the models on an independent external test set, thereby reducing potential bias introduced by overfitting. Moreover, the dataset used for training was, to the best of our knowledge, the largest MRI dataset of histopathologically confirmed lipomas and ALTs. In addition, a histopathological analysis was conducted by pathologists specialized in the analysis of soft-tissue tumors and included the immunohistochemistry for the assessment of the MDM2 status in all cases. Furthermore, we excluded inter-/intra-reader segmentation-dependent features and included variability features, making the model performance more stable and reliable for other datasets. 

## 5. Conclusions

In conclusion, radiogenomic models were developed that showed a high discriminatory power for predicting the MDM2 gene amplification status to distinguish between atypical lipomatous tumors and lipomas on preoperative MR images. The best-performing model was based on a LASSO algorithm using all MR sequences, with a higher accuracy compared to radiology residents, suggesting that these algorithms would be particularly helpful for radiologists with less experience. Due to the varying settings in which patients with lipomatous tumors present, this model may enhance the clinical diagnostic workup and improve the detection rate for atypical lipomatous tumors.

## Figures and Tables

**Table 1:** Patient characteristics.

Patient Characteristics	Internal Dataset (*n* = 257)	External Test Set (*n* = 50)
Age (years) *	62.4 ± 14.5	60.6 ± 12.5
Sex (women)	125	22
Tumor Location (Anatomical Region)		
Chest/Back	19	6
Neck	15	2
Leg	143	27
Arm	75	14
Hand	3	1
Foot	2	0
Lipomas	*n* = 192	*n* = 30
Age (years) *	62.3 ± 14.4	57.5 ± 11.1
Sex (women)	88	12
Atypical Lipomatous Tumors (ALT)	*n* = 65	*n* = 20
Age (years) *	62.5 ± 15	65.2 ± 13.5
Sex (women)	37	10

* Data are given as mean ± standard deviation.

**Table 2:** Performance of the machine-learning models on the external test set using demographic information or radiomic features only, as well as combining radiomic features and demographic information for the following model architectures: least absolute shrinkage and selection operator (LASSO), support vector machine (SVM), random forest classifier (RFC), and an artificial neural network (ANN). External performance represents the values yielded when a final cross-validation step considering only the best 150 best hyperparameter sets was implemented to predict the external test set.

Model Architecture	Score	Demographic Features	Combined Sequences	Combined Sequences + Demographic Features
LASSO	AUC *	0.56 (0.540.58) ± 0.07	0.88 (0.85–0.91) ± 0.07	0.72 (0.66–0.78) ± 0.15
	Accuracy	0.58	0.76	0.77
	Sensitivity	0.05	0.70	0.40
	Specificity	0.93	0.81	1.00
SVM	AUC *	0.54 (0.51–0.57) ± 0.12	0.84 (0.80–0.88) ± 0.11	0.85 (0.82–0.88) ± 0.09
	Accuracy	0.56	0.53	0.69
	Sensitivity	0.10	0.90	0.80
	Specificity	0.87	0.31	0.63
RFC	AUC *	0.63 (0.61–0.65) ± 0.06	0.87 (0.85–0.89) ± 0.05	0.87 (0.85–0.89) ± 0.05
	Accuracy	0.50	0.69	0.69
	Sensitivity	0.00	0.50	0.40
	Specificity	0.83	0.81	0.88
ANN	AUC *	0.68 (0.66–0.70) ± 0.08	0.81 (0.77–0.85) ± 0.10	0.81 (0.77–0.85) ± 0.10
	Accuracy	0.60	0.69	0.65
	Sensitivity	0.00	0.70	0.60
	Specificity	1.00	0.69	0.69

* Data are given as mean (95% confidence interval) ± standard deviation.

**Table 3:** Performance of the radiology residents with 2, 3, or 5 years of experience and the fellowship-trained radiologist that was experienced in musculoskeletal tumor imaging. Readers were blinded to all clinical and histopathological findings.

Score	Radiology Resident, 2y	Radiology Resident, 3y	Radiology Resident, 5y	Fellowship-Trained Radiologist
Accuracy	0.60 (30/50)	0.70 (35/50)	0.70 (35/50)	0.90 (45/50)
Sensitivity	0.55 (11/20)	0.60 (12/20)	0.80 (16/20)	0.96 (19/20)
Specificity	0.63 (19/30)	0.77 (23/30)	0.63 (19/30)	0.87 (26/30)

## Data Availability

The training parameters and source code can be found online (https://github.com/deedeedav/alt-lipoma-radiomics (accessed on 9 March 2023)).

## Supplementary Materials

The following supporting information can be downloaded at: https://www.mdpi.com/article/10.3390/cancers15072150/s1. Supplementary Material Table S1: Magnetic resonance imaging sequence parameters. Supplementary Material Table S2: Extracted radiomic features (*n* = 104). Supplementary Material Table S3: Performance of the machine-learning models on the external test set of each individual sequence (T1w, T2w, and T1fsgd) using the following model architectures: least absolute shrinkage and selection operator (LASSO), support vector machine (SVM), random forest classifier (RFC), and an artificial neural network (ANN). The external performance represents the values yielded when a final cross-validation step considering only the best 150 best hyperparameter sets was implemented. Supplementary Material Table S4: Internal performance representing the averaged values over 150 models resulting from the nested cross-validation using demographic information, radiomic features of each individual sequence (T1w, T2w, and T1fsgd), or radiomic features of all sequences combined, as well as combining radiomic features (of all sequences) and demographic information for the following model architectures: least absolute shrinkage and selection operator (LASSO), support vector machine (SVM), random forest classifier (RFC), and an artificial neural network (ANN). The metrics are given as mean ± standard deviation. Supplementary Material Table S5: Feature importance of the best-performing model (least absolute shrinkage and selection operator (LASSO) trained on features from all radiomic sequences). Supplementary Material Figure S1: Flow chart of the statistical analysis of the extracted radiomic features. Supplementary Material Figure S2: Confusion matrix of the best-performing model, a least absolute shrinkage and selection operator (LASSO) trained on all radiomic sequences. Misclassification rate: 0.23 ((FN + FP)/(N + P)). Supplementary Material Figure S3: Boxplot of the prediction probabilities made by the best-performing model (least absolute shrinkage and selection operator (LASSO) trained on features from all radiomic sequences). The probability cut-off used was 0.5.

## Abbreviations

atypical lipomatous tumors	ALTs
mouse double minute 2	MDM2
fluorescence in situ hybridization	FISH
turbo spin echo	TSE
volume of interest	VOI
intraclass correlation coefficient	ICC
principal component analysis	PCA
Neuroimaging Informatics Technology Initiative	NIfTI
support vector machine	SVM
random forest classifier	RFC
least absolute shrinkage and selection operator	LASSO
artificial neural network	ANN
area under the curve	AUC
receiver–operator curve	ROC
