# Supplementary material for: Development and Evaluation of MR-Based Radiogenomic Models to Differentiate Atypical Lipomatous Tumors from Lipomas
Source: Cancers (Basel). 2023 Apr 5;15(7):2150. doi: 10.3390/cancers15072150 (PMC10093205; doi:10.3390/cancers15072150)
Supplement: Supplementary file 1 [file cancers-15-02150-s001.zip › cancers-2197611-supplementary.pdf]

Supplemental Material

Sequence	T_1		T_2		T_1-FS-GD	
Field strength (T)	1.5	3.0	1.5	3.0	1.5	3.0
Repetition time (ms)	537-557	855-1100	4020-6110	4260-5600	500-595	722-1050
Echo time (ms)	15-16	12	85-104	78-94	13-16	12-13
Flip angle (°)	90-172	175-180	180	180	90-180	180-136
In-plane resolution (mm)	0.2-0.5x0.2-0.5	0.5-0.8x0.4-0.7	0.4-0.6x0.4-0.6	0.5-0.7x0.5-0.7	0.3-0.6x0.3-0.6	0.6-0.7x0.6-0.7
Slice thickness (mm)	3-4	3-5	3-5	4-5	3-5	4-5
Gap (%)	10-25	10-50	20-60	10-40	20-60	10-40
Bandwidth (Hz/pixel)	85-128	160-172	109-119	200-203	85-130	150-160
Echo train length (n)	118	162-262	14-58	15-37	118	116-288

GD: gadolinium-enhanced; FS: fat-saturated
Field of view was limited to the area of interest according to the respective tumor volume to maximize anatomical detail
Sequence parameters were adjusted in accordance with the optimal protocol of the anatomical tumor region

Supplementary Material Table S1: Magnetic Resonance imaging sequence parameters

Supplementary Material Table S2: Extracted radiomics features (n=104)

All extracted features were computed according the "image biomarker standardization initiative" (IBSI) guidelines [1].

	**Shape Features**
1	Mesh Volume
2	Voxel Volume
3	Surface Area
4	Surface Volume Ratio
5	Sphericity
6	Maximum 3D Diameter
7	Maximum 2D Diameter Slice
8	Maximum 2D Diameter Column
9	Maximum 2D Diameter Row
10	Major Axis
11	Minor Axis
12	Least Axis
13	Elongation
14	Flatness
	**First Order Features**
1	Energy
2	Intensity Histogram Entropy
3	Minimum
4	10th Percentile
5	90th Percentile
6	Maximum
7	Mean
8	Median
9	Interquartile Range
10	Range
11	Mean Absolute Deviation (MAD)
12	Root Mean Squared (RMS)
13	Skewness
14	Excess Kurtosis
15	Variance
16	Intensity Histogram Uniformity
	**Gray Level Co-occurrence Matrix (GLCM) Features**
1	Autocorrelation
2	Joint Average
3	Cluster Prominence
4	Cluster Shade
5	Cluster Tendency
6	Contrast
7	Correlation
8	Difference Average
9	Difference Entropy
10	Difference Variance

11	Joint Energy (IBSI: Angular Second Moment)
12	Joint Entropy
13	Informal Measure of Correlation (IMC) 1
14	Informal Measure of Correlation (IMC) 2
15	Inverse Difference Moment (IDM)
16	Inverse Difference Moment Normalized (IDMN)
17	Inverse Difference (ID)
18	Inverse Difference Normalized (IDN)
19	Inverse Variance
20	Maximum Probability (IBSI: Joint maximum)
21	Sum Entropy
22	Sum of Squares (IBSI: Sum of Squares)
23	Maximal Correlation Coefficient (MCC)
	**Gray Level Size Zone Matrix (GLSZM) Features**
1	Small Area Emphasis (SAE)
2	Large Area Emphasis (LAE)
3	Gray Level Non-Uniformity (GLN)
4	Gray Level Non-Uniformity Normalized (GLNN)
5	Size-Zone Non-Uniformity (SZN)
6	Size-Zone Non-Uniformity Normalized (SZNN)
7	Zone Percentage (ZP)
8	Gray Level Variance (GLV)
9	Zone Variance (ZV)
10	Zone Entropy (ZE)
11	Low Gray Level Zone Emphasis (LGLZE)
12	High Gray Level Zone Emphasis (HGLZE)
13	Small Area Low Gray Level Emphasis (SALGLE)
14	Small Area High Gray Level Emphasis (SAHGLE)
15	Large Area Low Gray Level Emphasis (LALGLE)
16	Large Area High Gray Level Emphasis (LAHGLE)
	**Gray Level Run Length Matrix (GLRLM) Features**
1	Short Run Emphasis (SRE)
2	Long Run Emphasis (LRE)
3	Gray Level Non-Uniformity (GLN)
4	Gray Level Non-Uniformity Normalized (GLNN)
5	Run Length Non-Uniformity (RLN)
6	Run Length Non-Uniformity Normalized (RLNN)
7	Run Percentage (RP)
8	Gray Level Variance (GLV)
9	Run Variance (RV)
10	Run Entropy (RE)
11	Low Gray Level Run Emphasis (LGLRE)
12	High Gray Level Run Emphasis (HGLRE)
13	Short Run Low Gray Level Emphasis (SRLGLE)
14	Short Run High Gray Level Emphasis (SRHGLE)
15	Long Run Low Gray Level Emphasis (LRLGLE)
16	Long Run High Gray Level Emphasis (LRHGLE)
	**Neighbouring Gray Tone Difference Matrix (NGTDM) Features**

1	Coarseness
2	Contrast
3	Busyness
4	Complexity
5	Strength
	**Gray Level Dependence Matrix (GLDM) Features**
1	Small Dependence Emphasis (SDE)
2	Large Dependence Emphasis (LDE)
3	Gray Level Non-Uniformity (GLN)
4	Dependence Non-Uniformity (DN)
5	Dependence Non-Uniformity Normalized (DNN)
6	Gray Level Variance (GLV)
7	Dependence Variance (DV)
8	Dependence Entropy (DE)
9	Low Gray Level Emphasis (LGLE)
10	High Gray Level Emphasis (HGLE)
11	Small Dependence Low Gray Level Emphasis (SDLGLE)
12	Small Dependence High Gray Level Emphasis (SDHGLE)
13	Large Dependence Low Gray Level Emphasis (LDLGLE)
14	Large Dependence High Gray Level Emphasis (LDHGLE)

[1] Zwanenburg, A.; Vallieres, M.; Abdalah, M.A.; Aerts, H.; Andrearczyk, V.; Apte, A.; Ashrafinia, S.; Bakas, S.; Beukinga, R.J.; Boellaard, R.; et al. The Image Biomarker Standardization Initiative: Standardized Quantitative Radiomics for High-Throughput Image-based Phenotyping. *Radiology* **2020**, *295*, 328–338. https://doi.org/10.1148/radiol.2020191145.

Supplementary Material Table S3: Performance the machine learning models on the external test set of each individual sequence T1w, T2w, and T1fsgd using the following model architectures: least absolute shrinkage and selection operator (LASSO), support vector machine (SVM), random forest classifier (RFC), and an artificial neural network (ANN). The external performance represents the values yielded when a final cross-validation step considering only the best 150 best hyperparameter sets was implemented.

Model Architecture	Score	T1w	T2w	T1fsgd
**LASSO**	**AUC***	0.83 (0.82-0.84) ± 0.02	0.82 (0.81-0.83) ± 0.04	0.84 (0.83-0.85) ± 0.03
	**Accuracy**	0.58	0.69	0.60
	**Sensitivity**	0.80	0.42	0.06
	**Specificity**	0.43	0.83	1.00
**SVM**	**AUC***	0.78 (0.76-0.80) ± 0.07	0.81 (0.78-0.84) ± 0.08	0.78 (0.74-0.82) ± 0.12
	**Accuracy**	0.64	0.63	0.65
	**Sensitivity**	0.30	0.17	0.24
	**Specificity**	0.89	0.88	0.96
**RFC**	**AUC***	0.80 (0.79-0.81) ± 0.02	0.80 (0.79-0.81) ± 0.04	0.82 (0.81-0.83) ± 0.04
	**Accuracy**	0.70	0.66	0.63
	**Sensitivity**	0.35	0.33	0.18
	**Specificity**	0.96	0.83	0.96
**ANN**	**AUC***	0.77 (0.75-0.79) ± 0.08	0.79 (0.76-0.82) ± 0.08	0.79 (0.77-0.81) ± 0.08
	**Accuracy**	0.71	0.58	0.70
	**Sensitivity**	0.45	0.08	0.29
	**Specificity**	0.89	0.83	1.00

* Data is given as mean (95% confidence interval) ± standard deviation

Supplementary Material Table S4: Internal performance representing the averaged values over 150 models resulting from the nested cross-validation using demographic information, radiomic features of each individual sequence T1w, T2w, and T1fsgd or radiomic features of all sequences combined, as well as combining radiomic features (of all sequences) and demographic information for the following model architectures: least absolute shrinkage and selection operator (LASSO), support vector machine (SVM), random forest classifier (RFC), and an artificial neural network (ANN). The metrics are given as mean ± standard deviation.

Model Architecture	Score	Demographic Features	T1w	T2w	T1fsgd	Combined Sequences	Combined Sequences + Demographic Features
**LASSO**	**AUC***	0.56 (0.55-0.57) ± 0.06	0.83 (0.83-0.83) ± 0.04	0.80 (0.79-0.81) ± 0.05	0.82 (0.81-0.83) ± 0.05	0.88 (0.87-0.89) ± 0.05	0.88 (0.87-0.89) ± 0.05
	**Accuracy***	0.67 (0.65-0.69) ± 0.15	0.79 (0.79-0.79) ± 0.04	0.78 (0.77-0.79) ± 0.05	0.79 (0.78-0.80) ± 0.04	0.85 (0.84-0.86) ± 0.04	0.85 (0.84-0.86) ± 0.05
	**Sensitivity**	0.14	0.41	0.34	0.34	0.51	0.49
	**Specificity**	0.85	0.92	0.91	0.92	0.93	0.94
**SVM**	**AUC***	0.56 (0.54-0.58) ± 0.12	0.80 (0.79-0.81) ± 0.09	0.78 (0.77-0.79) ± 0.08	0.75 (0.73-0.77) ± 0.13	0.84 (0.83-0.85) ± 0.09	0.85 (0.84-0.86) ± 0.08
	**Accuracy***	0.73 (0.73-0.73) ± 0.03	0.78 (0.78-0.78) ± 0.04	0.79 (0.78-0.80) ± 0.05	0.79 (0.78-0.80) ± 0.05	0.84 (0.83-0.85) ± 0.04	0.84 (0.83-0.85) ± 0.05
	**Sensitivity**	0.04	0.38	0.37	0.34	0.50	0.45
	**Specificity**	0.97	0.93	0.92	0.91	0.93	0.93
**RFC**	**AUC***	0.63 (0.62-0.64) ± 0.06	0.85 (0.84-0.86) ± 0.05	0.79 (0.78-0.80) ± 0.05	0.79 (0.78-0.80) ± 0.06	0.86 (0.85-0.87) ± 0.05	0.87 (0.86-0.88) ± 0.05
	**Accuracy***	0.69 (0.69-0.69) ± 0.04	0.81 (0.81-0.81) ± 0.03	0.80 (0.80-0.80) ± 0.03	0.80 (0.80-0.80) ± 0.03	0.86 (0.86-0.86) ± 0.03	0.86 (0.86-0.86) ± 0.03
	**Sensitivity**	0.23	0.50	0.39	0.36	0.49	0.51
	**Specificity**	0.85	0.92	0.92	0.92	0.95	0.95
**ANN**	**AUC***	0.68 (0.67-0.69) ± 0.08	0.78 (0.77-0.79) ± 0.08	0.76 (0.75-0.77) ± 0.09	0.77 (0.76-0.78) ± 0.08	0.83 (0.82-0.84) ± 0.08	0.79 (0.78-0.80) ± 0.09
	**Accuracy***	0.73 (0.73-0.73) ± 0.03	0.78 (0.77-0.79) ± 0.05	0.77 (0.76-0.78) ± 0.05	0.78 (0.77-0.79) ± 0.05	0.83 (0.82-0.84) ± 0.04	0.84 (0.83-0.85) ± 0.04
	**Sensitivity**	0.07	0.49	0.45	0.41	0.53	0.44
	**Specificity**	0.95	0.89	0.86	0.88	0.90	0.94

* Data is given as mean (95% confidence interval) ± standard deviation

Supplementary Material Table S5: Feature Importance of the best performing model (least absolute shrinkage and selection operator (LASSO) trained on features from all radiomic sequences).

Feature Name	Score
T1fs_original_glszm_ZoneEntropy	22,30362659

T1_original_shape_Elongation	21,75575404
T2_original_glszm_ZoneEntropy	19,1399285
T1fs_original_shape_Flatness	14,13490208
T1_original_shape_Maximum2DDiameterRow	14,06067563
T2_original_shape_Maximum2DDiameterRow	12,80611959
T1_original_ngtdm_Busyness	10,64687961
T2_original_shape_Maximum2DDiameterColumn	10,5844085
T1fs_original_firstorder_InterquartileRange	9,39608846
T2_original_shape_MajorAxisLength	9,064467759
T2_original_shape_Maximum3DDiameter	8,953856721
T1fs_original_firstorder_Energy	7,286263865
T1_original_shape_MajorAxisLength	6,747650604
T2_original_ngtdm_Complexity	2,597293753
T1_original_shape_Maximum2DDiameterSlice	2,586796401
T1fs_original_glcm_DifferenceEntropy	1,213683594
T1_original_shape_Maximum3DDiameter	1,118527794
T1fs_original_glcm_JointEntropy	0,253434882
T1_original_glszm_LargeAreaLowGrayLevelEmphasis	0,195501056
T1fs_original_glcm_JointAverage	0,11638087

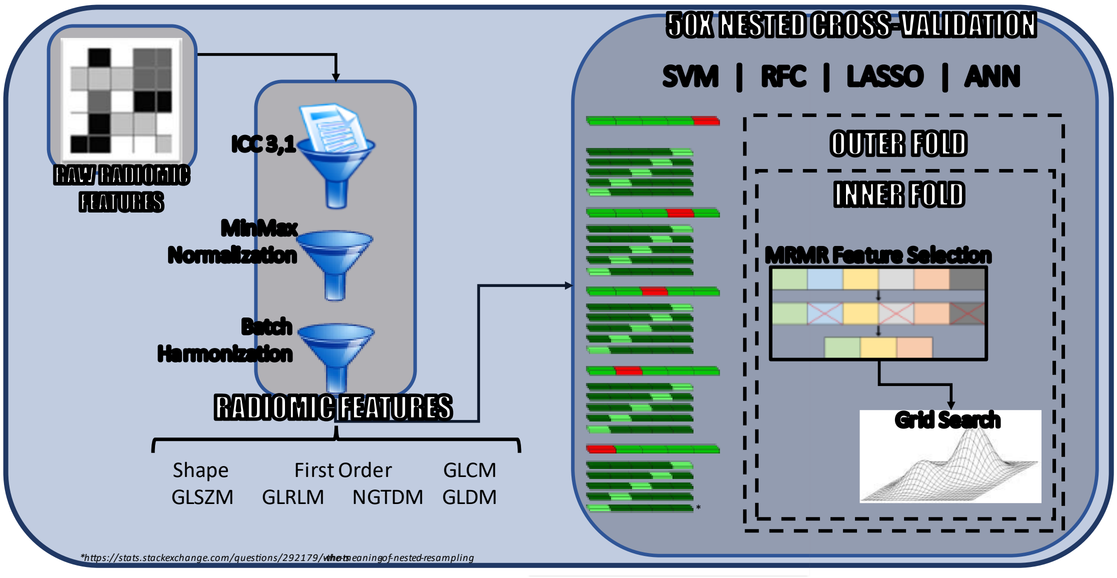

Supplementary Material Figure S1: Flow chart of the statistical analysis of the extracted radiomic features.

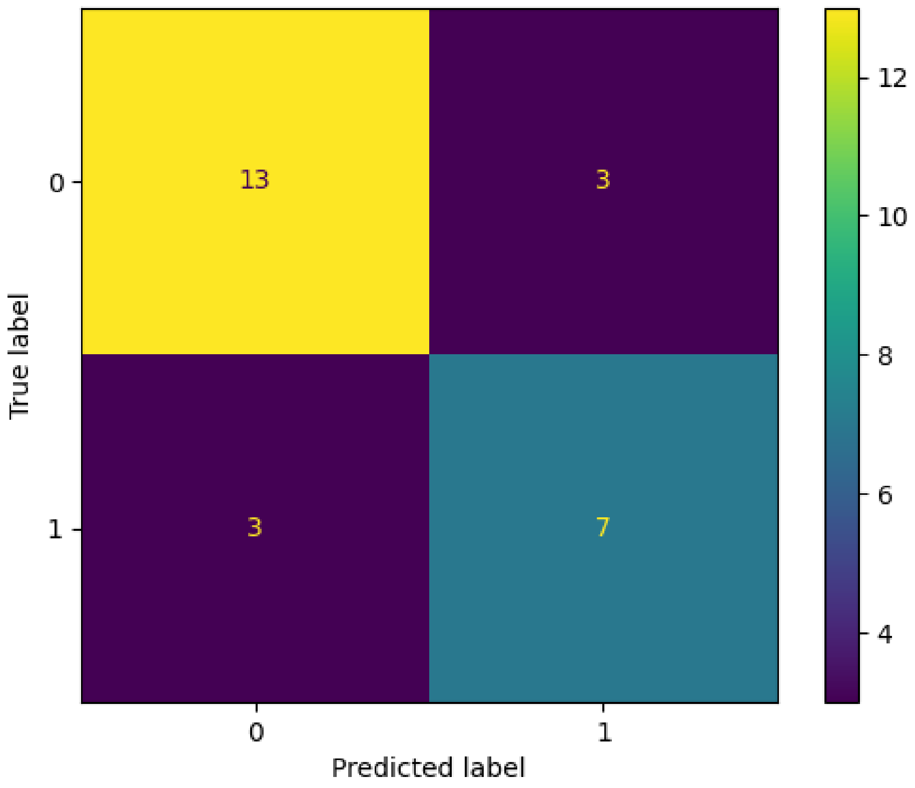

Supplementary Material Figure S2: Confusion matrix of the best performing model, a least absolute shrinkage and selection operator (LASSO) trained on all radiomic sequences. Misclassification rate: 0.23 ((FN + FP) / (N + P))

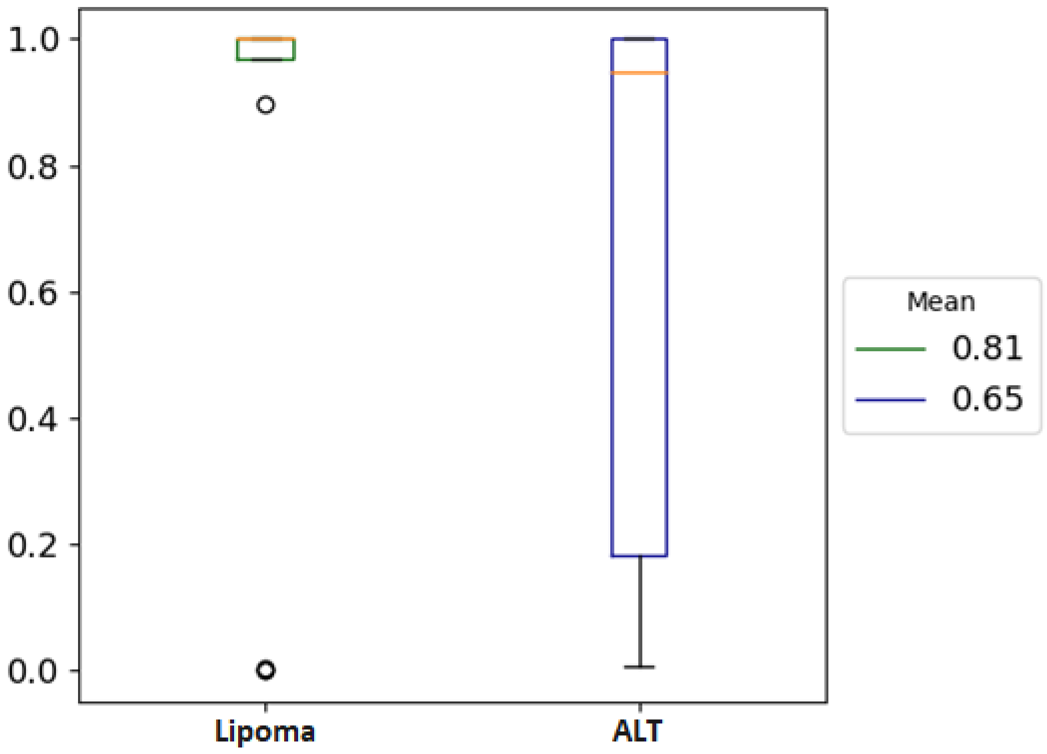

Supplementary Material Figure S3: Boxplot of the prediction probabilities made by the best performing model (least absolute shrinkage and selection operator (LASSO) trained on features from all radiomic sequences). The probability cut-off used was 0.5.
